# Human POLD1 modulates cell cycle progression and DNA damage repair

**DOI:** 10.1186/s12858-015-0044-7

**Published:** 2015-06-19

**Authors:** Jing Song, Ping Hong, Chengeng Liu, Yueqi Zhang, Jinling Wang, Peichang Wang

**Affiliations:** Department of Clinical Laboratory, Xuanwu Hospital Capital Medical University, No.45 Changchun Street, Xicheng District Beijing, 100053 China

**Keywords:** DNA polymerase delta, *POLD1*, Cell proliferation, Cell cycle, DNA damage

## Abstract

**Background:**

The activity of eukaryotic DNA polymerase delta (Pol δ) plays an essential role in genome stability through its effects on DNA replication and repair. The p125 catalytic subunit of Pol δ is encoded by *POLD1* gene in human cells. To clarify biological functions of POLD1, we investigated the effects of POLD1 overexpression or downregulation on cell proliferation, cell cycle progression, DNA synthesis and oxidative DNA damage induced by H_2_O_2_.

**Methods:**

HEK293 cells were transfected with POLD1 expression plasmid or shRNA, cell proliferation, cell cycle progression, and DNA synthesis in HEK293 cells were analyzed.

**Results:**

HEK293 cells were transfected with POLD1 expression plasmid or shRNA. POLD1 downregulation by shRNA suppressed cell proliferation, cell cycle progression, and DNA synthesis in HEK293 cells. However, POLD1 overexpression had no significant effects on these processes. Finally, comet assay showed that POLD1 downregulation led to increased DNA damage.

**Conclusions:**

Our results suggest that human POLD1 plays important role in the regulation of cell cycle progression and DNA damage repair.

## Background

Maintaining the integrity of DNA is dependent on the fidelity of DNA replication and is essential for the survival of cells and organisms. DNA polymerases are absolutely essential to the eukaryotic replication process [1]. DNA polymerase delta (Pol δ) plays an important role in genome maintenance through its involvement in replicative DNA synthesis and multiple synthetic repair processes [2]. DNA Pol δ has intrinsic 3’ to 5’exonuclease activity, which is fundamental to the function of the enzyme, and the interaction of DNA Pol δ with proliferating cell nuclear antigen (PCNA) allows it to replicate DNA processively [3].

Human DNA Pol δ is a multi-subunit complex comprised of four subunits: p125, p68, p50, and p12 [4]. The p125 subunit has been identified as the catalytic subunit and is encoded by the *POLD1* gene in human [5]. The polymerase and 3′-5′- exonuclease active sites of Pol δ reside in the p125 subunit [6]. Previous studies have shown that reducing the expression of the p125 subunit is sufficient to induce genomic instability, as reduced expression of the p125 subunit in yeast resulted in errors in DNA replication [7]. Another study linked lower expression of p125 subunit to fragile site instability in yeast, presumably by the induction of double-strand breaks at stalled replication forks [8]. Moreover, the age-related decrease in POLD1 expression has been shown to be involved in the classical DNA repair pathway in vitro [9]. Furthermore, these was an inverse correlation between POLD1 expression and age both in vivo and in vitro [10]. These data suggest that POLD1 may be associated with aging. However, how human POLD1 is involved in senescence-related processes remains unclear.

In the present study, we used HEK293 cells as the model to investigate the role of human POLD1 in senescence-related processes, including cell proliferation, cell cycle progression, DNA synthesis, and oxidative stress-induced DNA damage.

## Methods

### Cell culture

HeLa cells and HEK293 cells were purchased from Shanghai Cell Institute Country Cell Bank. All cells were cultured in Dulbecco’s modified Eagle’s medium (DMEM, Life Technologies, USA) supplemented with 10 % fetal bovine serum (FBS, Life Technologies) in a humidified incubator with 5 % CO_2_ at 37 °C. The medium was replaced every 2 days, and cells were passaged once. Exponentially growing cells were selected for the experiments.

### Plasmids and shRNAs

To construct a plasmid for the overexpression of POLD1, *POLD1* cDNA was isolated from HeLa cells. The full-length *POLD1* cDNA (GenBank accession number M80397) was amplified by polymerase chain reaction (PCR) using the following primers: 5’-CGCGGATCCCTGTGGCGGGAAACGCTGTTTGAAG-3’ and 5’- CAACAAGCTTCAAGGTCACCAGGCCTCAGGTCCAG-3’, and subcloned into pcDNA3.0 to construct pcDNA3.0-POLD1. Positive clones were confirmed by DNA sequencing.

Short hairpin RNA (shRNA) targeting POLD1 (shPOLD1) and the negative control shRNA (shControl) were purchased from BGI (Shenzhen, China). The oligonucleotides encoding POLD1 shRNA were as follows: 5’-CACCGCTTCGCTCCCTACTTCTACACGAATGTAGAAGTAGGGAGCGAAGC-3’ and 5’-AAAAGCTTCGCTCCCTACTTCTACATTCGTGTAGAAGTAGGGAGCGAAGC-3’.

### Transient transfection

HEK293 cells were seeded in 6-well culture plates 1 day before transfection. POLD1 plasmid and shRNA as well as negative controls were transfected into HEK293 cells using Lipofectamine 2000 (Life Technologies) according to the manufacturer’s instructions. At 48–72 h after transfection, the cells were collected for further analysis.

### Quantitative real-time reverse transcription PCR

Total RNA was isolated from cells using a UNIQ-10 Column Total RNA Purification Kit (Sangon Biotech, Shanghai, China) and quantified using a NanoDrop 2000 UV–vis spectrophotometer (Thermo Fisher Scientific, USA). RNA was reverse transcribed using a One Step PrimeScript cDNA Synthesis kit (Takara, Japan). The POLD1 sense primer was 5′- CAACCTGGTCACTGCCTCAC-3′, and the antisense primer was 5′- GTCCCGCTTCCTCATCCTCT-3′. For the β-actin gene, the sense primer was 5′-GCTCAGGAGGAGCAATGATCTTG-3′, and the antisense primer was 5′-GTACGCCAACACAGTGCTGTC-3′. Real-time PCR analysis was performed in an ABI 7500 FAST Real-Time PCR System (Applied Biosystems, CA, USA) using SYBR Green (Takara). Relative expression levels were calculated using the 2^−ΔΔCt^ method and quantified after normalization to β-actin. Each experiment was performed in triplicate and repeated three times.

### Western blot analysis

Cells were lysed in RIPA lysis buffer (Beyotime, Nanjing, China) containing the protease inhibitor phenylmethanesulfonyl fluoride (Beyotime). Protein concentrations were determined using a BCA Protein Assay kit (Tiangen, Beijing, China). Equal amounts of protein were loaded on polyacrylamide gels and separated by sodium dodecyl sulfate-polyacrylamide gel electrophoresis. Proteins were then transferred to polyvinylidene fluoride membranes (Millipore, Billerica, MA, USA). Membranes were blocked with 5 % w/v nonfat dry milk in Tris-buffered saline containing Tween 20 (TBST: 20 mM Tris–HCl pH7.8, 150 mM NaCl, and 0.05 % Tween 20) for 1 h at room temperature. The blots were then incubated with antibodies against POLD1 (1:1000 dilution; Abcam, UK) and GAPDH (1:2000 dilution; GenScript, USA) at 4 °C overnight. After incubation with the corresponding horseradish peroxidase (HRP)-conjugated secondary antibodies, protein bands were visualized using a SuperSignal West Pico kit (Thermo Fisher Scientific). Densitometric analysis of protein bands was performed using Image Lab software (Bio-Rad, Hercules, CA, USA).

### Cell proliferation assay

The proliferation of HEK293 was assessed using a Cell Counting Kit-8 detection kit (CCK-8; Beyotime). Briefly, cells were seeded in triplicate in 96-well plates at 5 × 10^3^ cells/well. Adherent cells were transfected with pcDNA3.0-POLD1, pcDNA3.0, shPOLD1, or shControl. At 24, 48, and 72 h after transfection, 10 μL CCK-8 solution was added to each well, and plates were then incubated for 2 h at 37 °C. Absorbance values of all wells were then determined at 450 nm in a Microplate Reader (Thermo Fisher Scientific).

### Cell cycle analysis

Cell cycle was analyzed using flow cytometry with propidium iodide (PI) staining. Briefly, 48 h after transfection, HEK293 cells (2 × 10^6^ for each sample) were harvested in triplicate. After washing twice with cold phosphate-buffered saline (PBS), the cells were resuspended in 70 % ethanol and fixed overnight at 4 °C. Next, the fixed cells were washed with PBS and incubated with RNase A and PI at 37 °C for 30 min in the dark. The cell cycle distribution was then analyzed by flow cytometry (BD Biosciences, USA).

### 5-Ethynyl-2′-deoxyuridine (EdU) incorporation assay

DNA synthesis was assessed by the incubation of cells with 10 μM EdU for 30 min, followed by staining according to the manufacturer’s recommendations (RiboBio, Guangzhou, China). Cells were then harvested and analyzed by flow cytometry (BD Biosciences).

### Comet assay

At 48 h after transfection, HEK293 cells were treated with 150 μM H_2_O_2_ for 5 min at 4 °C in the dark. Cells were then washed with PBS and immediately analyzed using comet assay as previously described [9]. Data were analyzed using CASP software (CASP-1.2.2), and the Olive tail moment (OTM) was measured.

### Statistical analysis

Statistical analysis was performed using SPSS 13.0 software (SPSS Inc., Chicago, IL, USA). Values were presented as means ± standards error (SEs). Comparisons between groups were performed by one-way analysis of variance (ANOVA) followed by post-hoc Tukey analysis. *P* values of less than 0.05 were considered statistically significant.

## Results

### Overexpression or downregulation of POLD1 in HEK293 cells

**Fig. 1:**
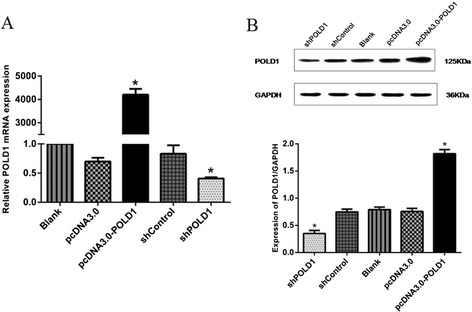
Modulation of POLD1 expression in HEK293 cells. **a** qRT-PCR analysis of POLD1 mRNA level in HEK293 cells transfected with pcDNA3.0-POLD1, pcDNA3.0, shPOLD1 or shControl. **b** Western blot analysis of POLD1 protein level in HEK293 cells transfected with pcDNA3.0-POLD1, pcDNA3.0, shPOLD1 or shControl. Untransfected HEK293 cells were used as a control (Blank). n = 3, *P < 0.01 vs. the negative control (pcDNA3.0 or shControl)

First, we introduced POLD1 expression vector or POLD1 shRNA into HEK293 cells, using pcDNA3.0 and shControl plasmids as negative controls. qRT-PCR analysis demonstrated that POLD1 mRNA level was increased over 4000-fold in HEK293 cells transfected with pcDNA3.0-POLD1, compared to control (Fig. 1a). Conversely, POLD1 mRNA level was decreased by 50 % following transfection with shPOLD1, compared to control (Fig. 1a). Western blot analysis confirmed that POLD1 protein level was significantly increased following transfection with pcDNA3.0-POLD1 (*P* < 0.01), but was significantly downregulated following transfection with shPOLD1 (*P* < 0.01), compared with the respective negative controls (Fig. 1b). Taken together, these data indicate that using plasmids or shRNA we constructed, we could effectively overexpress or downregulate POLD1 in HEK293 cells.

### POLD1 shRNA inhibits the proliferation of HEK293 cells

**Fig. 2:**
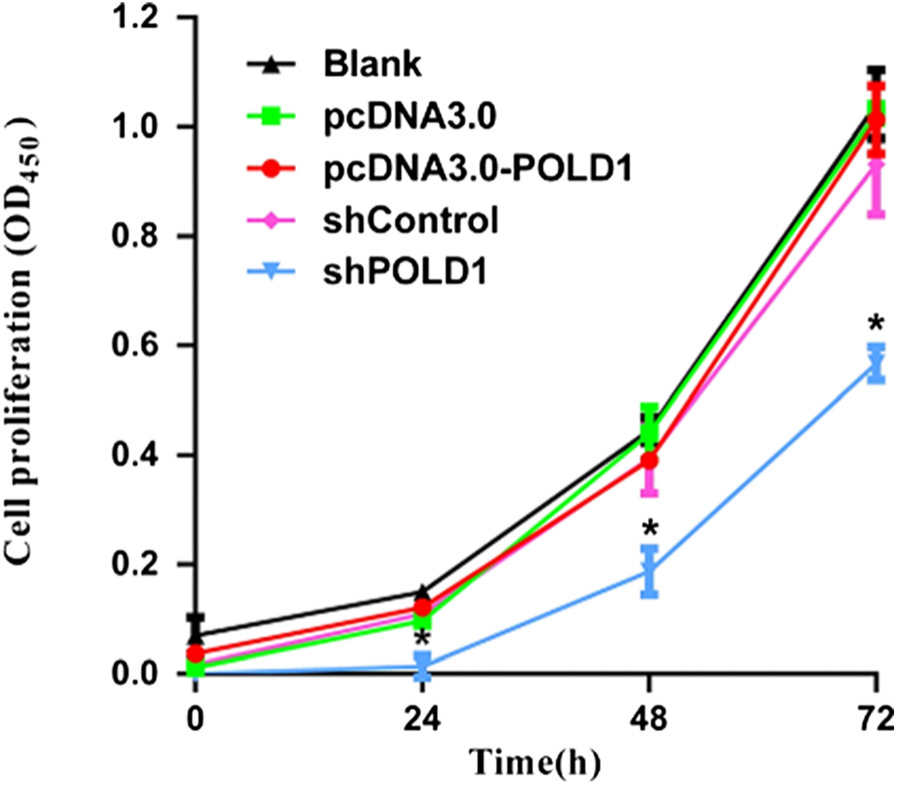
Effects of altered POLD1 expression on the growth of HEK293 cells. HEK293 cells were transfected with the indicated vectors, and cell proliferation was determined by CCK-8 assay 24, 48, and 72 h later. Data shown were the mean ± SD of the ratio for the absorbance at 450 nm. n = 3, *P < 0.01 vs. shControl

CCK-8 assay showed that the proliferation of HEK293 cells was significantly inhibited after transfection with shPOLD1 compared to cells transfected with shControl (*P* < 0.01). However, overexpression of POLD1 did not significantly alter cell proliferation compared with the control (*P* > 0.05) (Fig. 2).

### POLD1 shRNA leads to cell cycle arrest in HEK293 cells

**Fig. 3:**
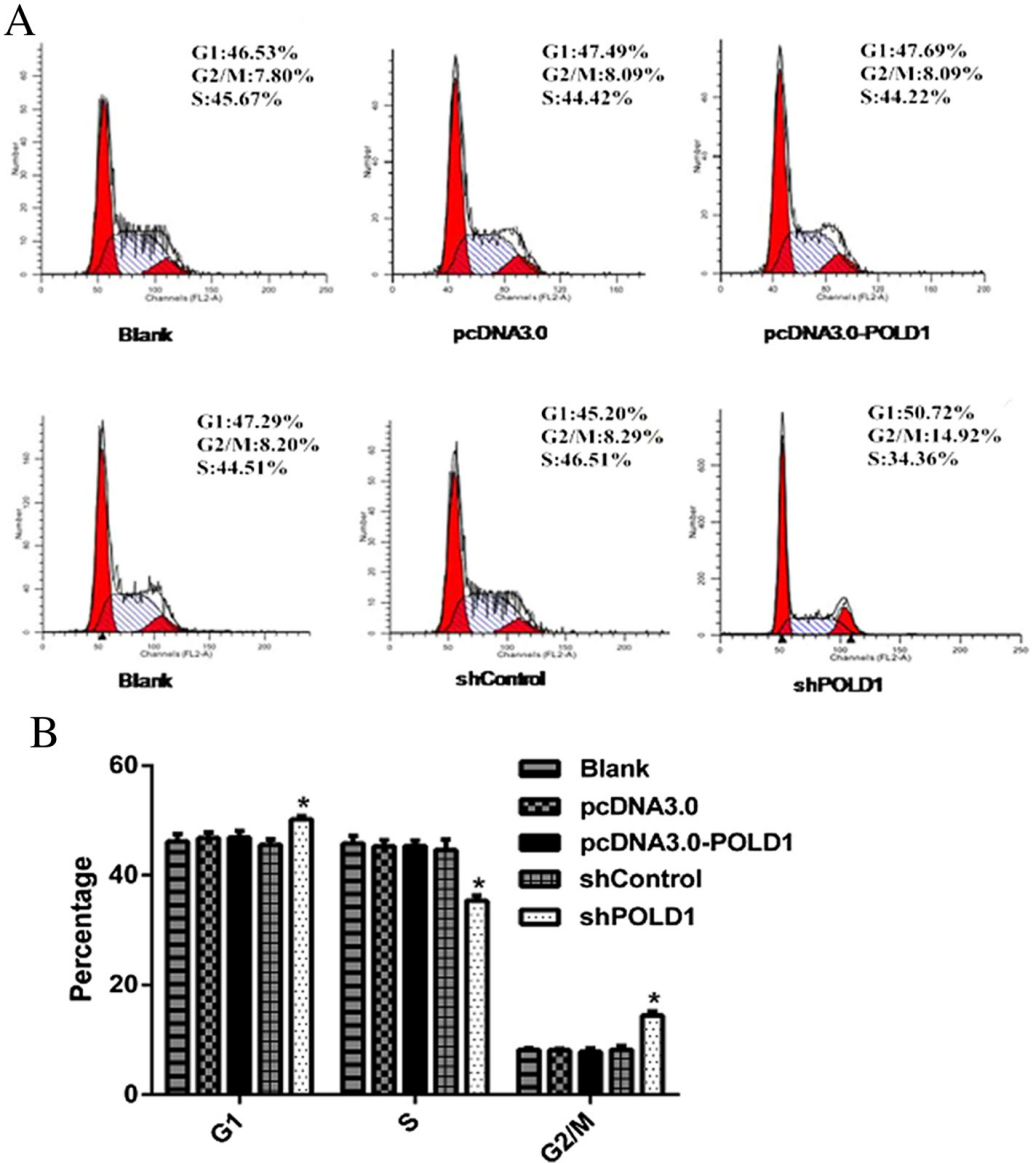
Effects of altered POLD1 expression on e cell cycle progression of HEK293 cells. **a** Representative FACS plots showing cell cycle distribution of HEK293 cells transfected with the indicated vectors. **b** Quantification of the percentage of cells in different phases. n = 3, *P < 0.01 vs. shControl

Since POLD1 shRNA suppressed the proliferation of HEK293 cells, we wondered whether these changes are associated with the regulation of cell cycle progression. Thus, we analyzed cell cycle distribution by flow cytometry analysis in HEK293 cells following transient transfection with pcDNA3.0-POLD1, pcDNA3.0, shPOLD1, or shControl. We found that the percentages of cells in G1 and G2/M phases were increased after transfection with shPOLD1, compared to the control. Although the increases were small, they showed significant differences (*P* < 0.05). However, overexpression of POLD1 by transfection with pcDNA3.0-POLD1 did not affect the cell cycle distribution compared to the control (Fig. 3). Taken together, these results demonstrate that POLD1 shRNA inhibits cell cycle at both G1 and G2/M phases in HEK293 cells.

### POLD1 shRNA reduces the rate of DNA synthesis in HEK293 cells

**Fig. 4:**
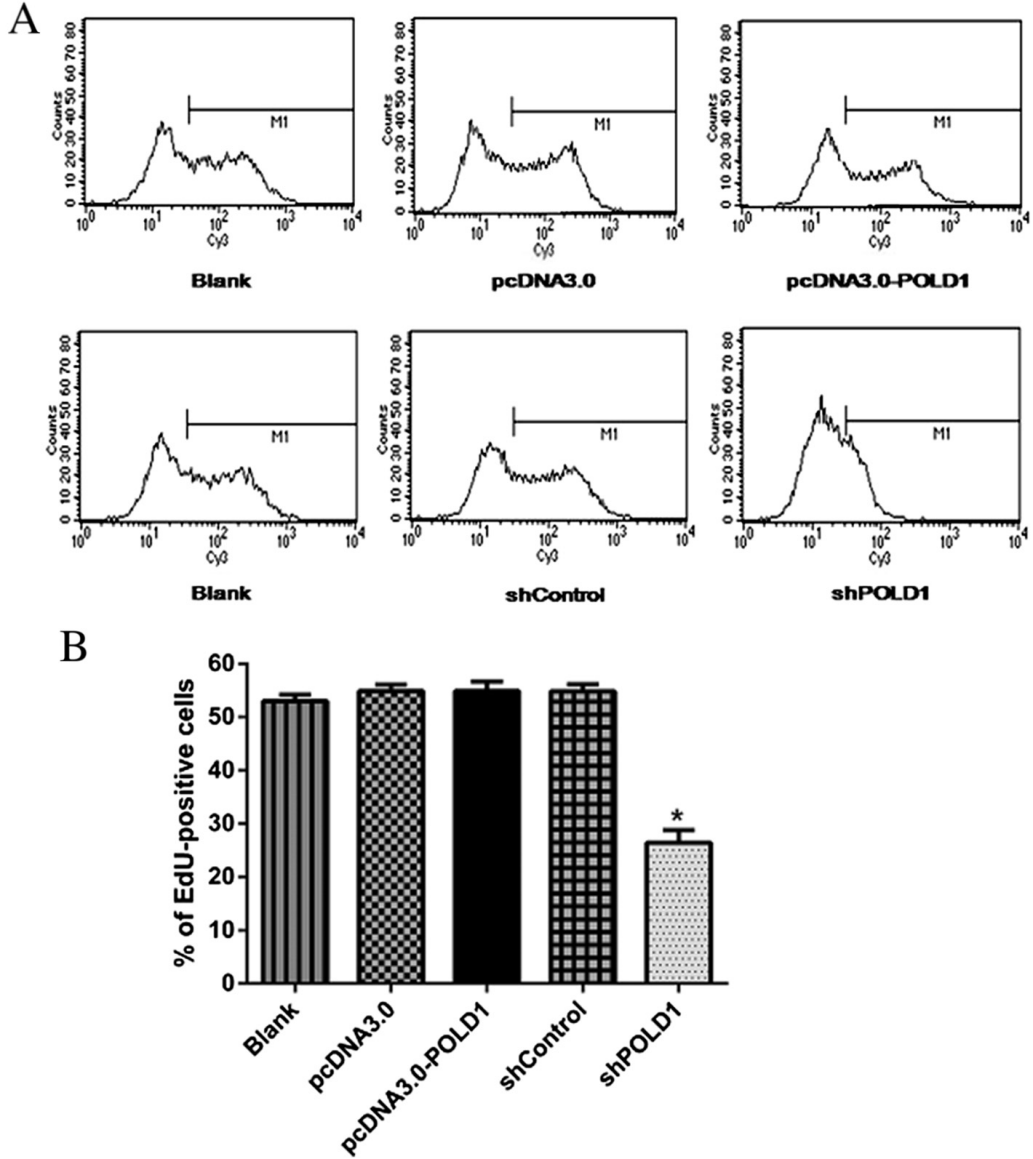
Quantification of DNA synthesis rate in HEK293 cells. **a** HEK293 cells were transfected with the indicated vectors, 48 h after transfection EdU-positive cells were determined by flow cytometry. **b** Quantification of the percentage of cells positive for EdU incorporation assay shown in (**a**). n = 3, *P < 0.01 vs. shControl

To further investigate the role of POLD1 in HEK293 cell proliferation, we performed EdU incorporation assay. Compared with shControl-transfected cells, shPOLD1-transfected cells exhibited a 30 % reduction in the rate of EdU incorporation. Consistent with the results of cell cycle analysis, there was no significant difference in DNA synthesis following transfection with pcDNA3.0-POLD1 compared with the control (Fig. 4). Collectively, these data suggest that POLD1 downregulation leads to attenuated DNA synthesis in HEK293 cells.

### POLD1 shRNA increases oxidative DNA damage in HEK293 cells

**Fig. 5:**
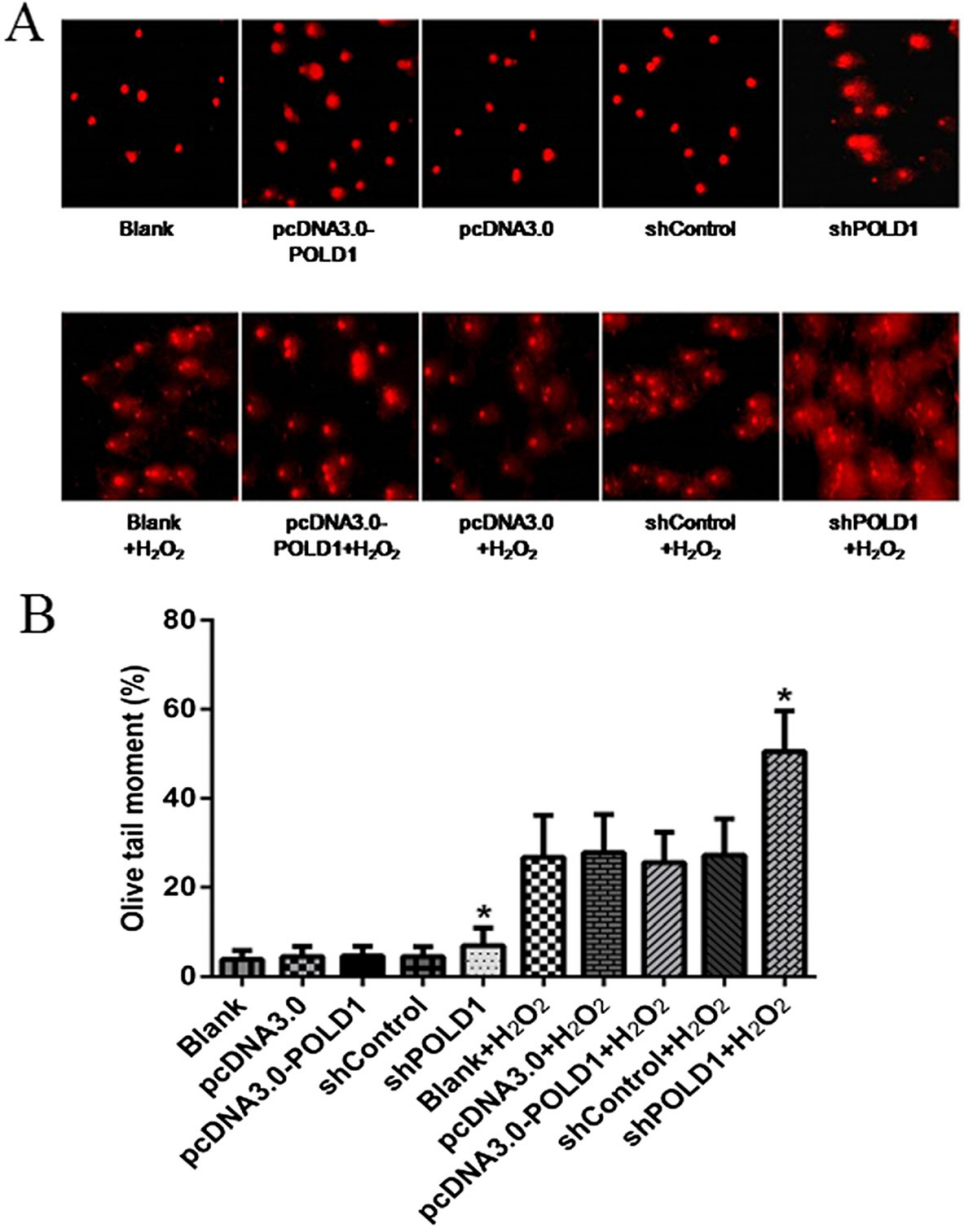
Effects of altered POLD1 expression on DNA damage in HEK293 cells. **a** HEK293 cells were transfected with the indicated vectors and treated with 150 μM H_2_O_2_ for 5 min in the dark. Comet assay was then performed, and images were acquired using a fluorescence microscope to show DNA fragment migration patterns. **b** Quantification of the tail moments from the comet assay shown in (**a**). *P < 0.01, vs. the negative control (shControl or shControl + H_2_O_2_)

Since DNA Pol δ plays important roles in DNA damage repair, we examined whether POLD1 could protect HEK293 cells against oxidative DNA damage. By comet assay we found that there were no differences in DNA damage among HEK293 cells transfected with pcDNA3.0-POLD1 or pcDNA3.0, after treatment with 150 μM H_2_O_2_. However, POLD1 shRNA led to increased DNA damage in HEK293 cells after treatment with 150 μM H_2_O_2_, compared to control shRNA transfected cells (Fig. 5). These data suggest that POLD1 shRNA impairs DNA damage repair in HEK293 cells.

## Discussion

Cell proliferation plays an essential role in senescence [11, 12]. In the present study, we found that the downregulation of *POLD1* gene, which encodes the catalytic subunit of DNA Pol δ, markedly inhibited cell proliferation. Further analysis showed that the inhibition of cell proliferation following POLD1 downregulation was related to the inhibition of cell cycle progression and DNA synthesis. Interestingly, we found that POLD1 overexpression had no significant effects on cell proliferation, cell cycle progression and DNA synthesis. We think that endogenous POLD1 may be enough to regulate these processes and exogenous overexpression of POLD1 could not achieve additional effects. Collectively, our data suggest that POLD1 promotes cell proliferation by regulating cell cycle and DNA synthesis.

In mammals, cell proliferation and cell cycle progression are tightly regulated processes, and loss of expression of specific genes encoding proteins essential for DNA synthesis may lead to mitotic dysregulation in senescent cells [13]. POLD1 is a key component of DNA Pol δ catalytic subunit and plays an important role in cell growth and differentiation [14]. Previous studies have indicated that replicatively senescent cell populations arrest in both G1and G2 stages [15, 16]. In our study, we found that POLD1 shRNA blocked cell cycle at G1 and G2/M phases and resulted in reduced DNA synthesis. These results provide new evidence for the potential role of POLD1 in the regulation of cell cycle progression. Studies have shown that the *POLD1* promoter has consensus sequences at the Sp1 and E2F binding sites [6]. E2F is thought to be crucial for the G1/S transition and DNA replication. Therefore, E2F may regulate POLD1 expression during cell cycle progression. Moreover, p53 represses Sp1-stimulated POLD1 promoter activity [17, 18], and p21 blocks E2F1 release by binding to the POLD1 promoter at the E2F1 binding site, thereby inhibiting POLD1 activity [19]. These findings may explain the mechanisms by which POLD1 is involved in the regulation of cell proliferation.

During aging, free radicals and reactive oxygen species (ROS) accumulate in cells and tissues. ROS generate a variety of DNA lesions, including oxidized DNA bases, abasic sites, single-strand breaks (SSBs), and double-strand breaks (DSBs) [20]. Previous data indicate that oxidative stress appears to play an essential role in the process of premature aging [21]. Therefore, efficient DNA repair in is very important to prevent cell aging. The predominant repair pathways in mammalian cells are base excision repair (BER), nucleotide excision repair, DSB repair and mismatch repair [22, 23]. BER is a major mechanism involved in the protection of cells from mutagenic base damage spontaneously generated through normal cellular metabolism or DNA damage caused by exogenous agents, such as oxidative stress, hydrolysis, and environmental factors. In addition to its crucial role in DNA replication, DNA Pol δ plays an essential role in DNA repair and is generally regarded as the primary enzyme involved in resynthesis (gap filling) in various DNA repair processes [24]. DNA Pol δ plays an important role by modulating the rate of single-nucleotide BER and long patch BER during the repair process [25]. Therefore, we hypothesized that POLD1 could affect oxidative DNA damage in HEK293 cells. Our results showed that POLD1 shRNA markedly resulted in increased DNA damage induced by H_2_O_2_, potentially through inhibiting DNA replication (leading to elevated DSBs) and DNA synthesis associated with DNA repair. Our data suggest that POLD1 may provide protection against oxidative DNA damage through various DNA repair systems.

However, secondary roles of POLD1 in other pathways should be considered. It is possible that some unidentified factors are activated following POLD1 downregulation and contribute to the inhibition of cell proliferation and increased DNA damage. Further studies are needed to elucidate the mechanisms by which POLD1 regulates cell cycle and DNA damage response.

## Conclusions

In summary, we demonstrated that POLD1 downregulation markedly inhibited cell proliferation by regulating cell cycle and DNA synthesis. POLD1 downregulation also resulted in increased DNA damage induced by H_2_O_2_. Therefore, POLD1 plays an important biological role in cell cycle regulation and DNA damage repair.

## Abbreviations

shRNA: Short hairpin RNA
ROS: Reactive oxygen species
SSBs: Single-strand breaks
DSBs: Double-strand breaks
